# Supplementary material for: Attenuating Effects of Pyrogallol-Phloroglucinol-6,6-Bieckol on Vascular Smooth Muscle Cell Phenotype Changes to Osteoblastic Cells and Vascular Calcification Induced by High Fat Diet
Source: Nutrients. 2020 Sep 11;12(9):2777. doi: 10.3390/nu12092777 (PMC7551448; doi:10.3390/nu12092777)
Supplement: Supplementary file 1 [file nutrients-12-02777-s001.pdf]

Supplementary material

Table S1. List of primer for real-time quantitative real time polymerase chain reaction (qRT-PCR).

Gene name (Gene ID)		Primer sequence
Actb	Forward	5' - ACA AAG CTG TTC AGT GTC TCC A - 3′
**(11461)**	Reverse	5' - CTC CGT TTC CAG AAT ACA CAC A- 3′
PKC	Forward	5′ - GAT CTC CAT CGG ACT GTT CTT C - 3'
(18752)	Reverse	5′ - ATT TTG ATG TGC CCT TCT GAG T- 3'
TGFβ	Forward	5' - CTG GCA GTA GCT CCC CTA TTT A-3′
(21808)	Reverse	5' - ACC AGG GTA AAA ATC GAG ATG A-3′
BMP2	Forward	5′ - ACA GTA GTT TCC AGC ACC GAA T- 3'
(12156)	Reverse	5′ - TGT CCA ATA GTC TGG TCA CAG G- 3'
NF-κB	Forward	5' - AGA AAT CCT ACC CAC AGG TCA A-3′
(18033)	Reverse	5' - CAT TTG TGA CCA ACT GAA CGA T-3′
CBFα1	Forward	5′ - CGG GAA TGA TGA GAA CTA CTC C- 3'
(12393)	Reverse	5′ - TTG AAC CTG GCT ACT TGG TTT T- 3'
osteocalcin	Forward	5′ - GTG TGA GCT TAA CCC TGC TTG T- 3'
(12097)	Reverse	5′ - AGT GAT ACC GTA GAT GCG TTT G- 3'

Supplementary methods

Alkaline phosphatase (ALP) activity assay

The Alkaline phosphatase (ALP) activity was determined in mouse serum using ALP activity assay kit (Abcam, UK). A quantity of 80 µl of serum was reacted with 50 µl of the 5 mM pnitrophenyl phosphate as the substrate for 60-minute before ending the reaction with the stop solution. After that the absorbance of sample was measured at 405 nm on a microplate reader.

Calcium deposition assay

Calcium quantification was measured by using a commercial kit (Abcam, UK). A quantity of 50 µl of serum sample was transferred into new wells. Chromogenic buffer was added and incubated for 5-minute. After that the absorbance of sample was detected at 575 nm on a microplate reader.

Alizarin red S staining

To validate calcium in aorta tissue, 0.2% alizarine red S staining conducted. The depariffinized slides washed with distilled water twice and then the slide incubated with 0.2% alizarine red S incubated for 5-minute and dehydrated in acetone. The stained slides imgersed with hematoxylin for nuclei counting and cleared with xylene and mounted with mounting medium (Sigma-Aldrich).

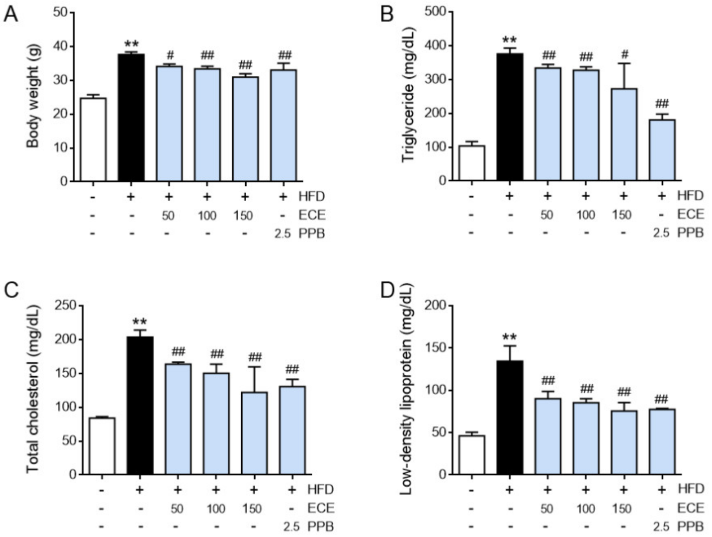

Figure S1. Changes of body weight, triglyceride, total cholesterol and LDL level of HFD-fed mice.C57BL/6N mice (male, 8 weeks of age) were bought and NFD or HFD-fed for 4 weeks and then 0.9% normal saline orally administered with NFD (NFD group) or HFD (HFD group) for the last 4 weeks. ECE (ECE group) of PPB (PPB group) orally administered with HFD for the last 4 weeks. At the end of the 8-week study period, (**A**) body weight measured and then all mice were sacrificed. **(B)** triglyceride, (**C**) total cholesterol and (**D**) LDL level measured in blood serum of mice. **, $p < 0.01$ vs. NFD/Saline group; #, $p < 0.05$, ##, $p < 0.01$ vs. HFD/Saline group. ECE, *Ecklonia cava* extract; HFD, high-fat diet; LDL, low-density lipoproteins; NFD, normal fat diet; PPB, pyrogallol-phloroglucinol-6,6-bieckol.

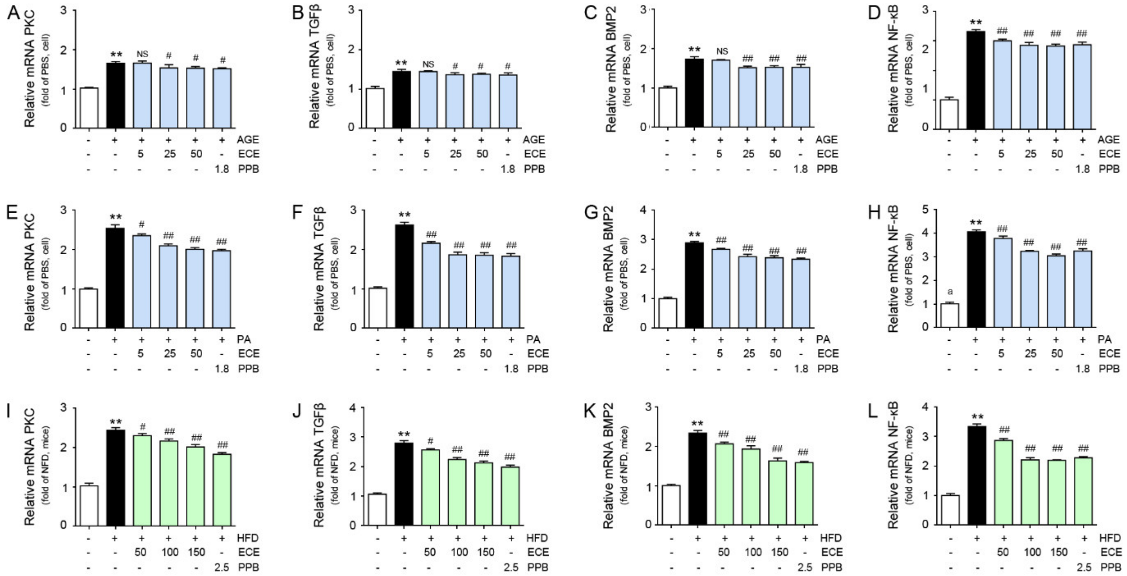

Figure S2. Inhibitory effects of ECE or PPB administration on vascular smooth muscle cell phenotypic switching to osteoblast-like cells mRNA levels of the phenotypic switching to osteoblast-like cell-related molecules PKC (**A, E, I**), TGFβ (**B,F,J**), BMP2 (**C,G,K**), NF-κB (**D,H,L**) were determined using qRT-PCR. 0, 25,50 ug/ml ECE or 1.8 ug/ml PPB were incubated with AGE or PA-treated VSMC (cell) and 50, 100, 250 mg/kg ECE or 2.5 mg/kg PPB administered with HFD-fed mice. **, $p < 0.01$ vs. PBS or NFD/Saline group; #, $p < 0.05$, ##, $p < 0.01$ vs. AGE, PA or HFD/Saline group. AGE, advanced glycation end products; BMP2, bone morphogenetic protein 2; ECE, *Ecklonia cava* extract; HFD, high-fat diet; NFD, normal fat diet; NF-κB, nuclear factor kappa-light-chain-enhancer of activated B cells; NS, non-

signal; PA, palmitate; PKC, protein kinase C; PPB, pyrogallol-phloroglucinol-6,6-bieckol; qRT-PCR, real-time quantitative real time polymerase chain reaction; TGFβ, transforming growth factor beta; VSMC, vascular smooth muscle cell.

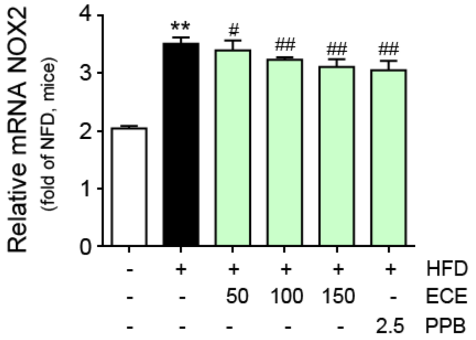

Figure S3. Inhibitory effects of ECE or PPB administration on NOX2 expression**.** mRNA levels of NOX2 was determined using qRT-PCR. 50, 100, 150 mg/kg ECE or 2.5 mg/kg PPB were administrated with HFD-fed mice. The asterisk (*) indicates difference between some groups vs. NFD group and the sharp (#) indicates difference between some groups vs. HFD group. **, $p < 0.01$ vs. NFD/Saline group; #, $p < 0.05$, ##, $p < 0.01$ vs. HFD/Saline group. ECE, *Ecklonia cava* extract; HFD, high-fat diet; NFD, normal fat diet; NOX2, nicotinamide adenine dinucleotide phosphate oxidase 2; PPB, pyrogallol-phloroglucinol-6,6-bieckol; qRT-PCR, real-time quantitative real time polymerase chain reaction.

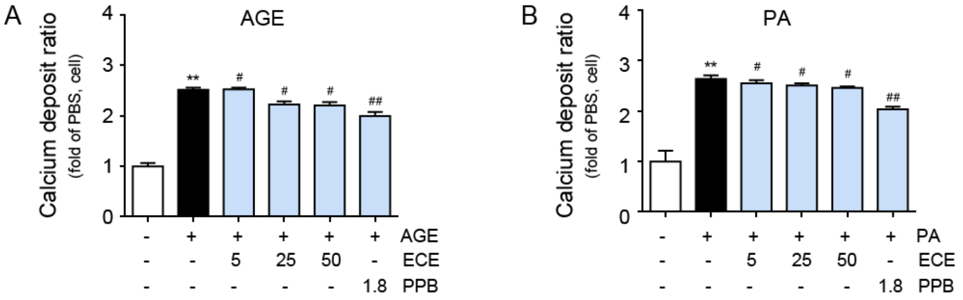

Figure S4. Inhibitory effects of ECE or PPB administration on calcium deposition of vascular smooth muscle cells. 0, 25,50 ug/ml ECE or 1.8 ug/ml PPB were incubated with AGE (A) or PA-treated VSMC (B) and Calcium deposition was calculated using the calcium deposition assay. **, $p < 0.01$ vs. PBS group; #, $p < 0.05$, ##, $p < 0.01$ vs. AGE or PA or group. AGE, advanced glycation end products; ECE, *Ecklonia cava* extract; PA, palmitate; PPB, pyrogallol-phloroglucinol-6,6-bieckol; VSMC, vascular smooth muscle cell.
